# Digest: Following clines along an Amazonian hybrid zone^*^

**DOI:** 10.1111/evo.14437

**Published:** 2022-02-01

**Authors:** Jente Ottenburghs

**Affiliations:** ^1^ Wildlife Ecology and Conservation Wageningen University Wageningen 6708 PB The Netherlands; ^2^ Forest Ecology and Forest Management Wageningen University Wageningen 6708 PB The Netherlands

## Abstract

The shape and position of clines can provide crucial insights into the evolutionary forces at work in hybrid zones. In this issue, Del‐Rio and colleagues applied cline theory to a hybrid zone between two antbird species in Amazonia. A narrow and displaced mitochondrial cline suggests that the selected genetic marker failed to track the northward movement of this hybrid zone, possibly due to reduced fitness of female hybrids.

Cline theory provides a powerful conceptual framework to analyze hybrid zone dynamics (Gompert et al. 2017). By comparing the shape and position of different clines—based on morphological traits or molecular markers—researchers can make inferences about biological features, such as hybrid fitness. A hypothetical example helps to illustrate the basics of cline theory (Fig. 1). Imagine a white and a black bird species that produce gray offspring in a hybrid zone. Tracking their plumage color along a geographical transect will reveal a clinal transition from white, to gray, to black birds.

**Figure 1:**
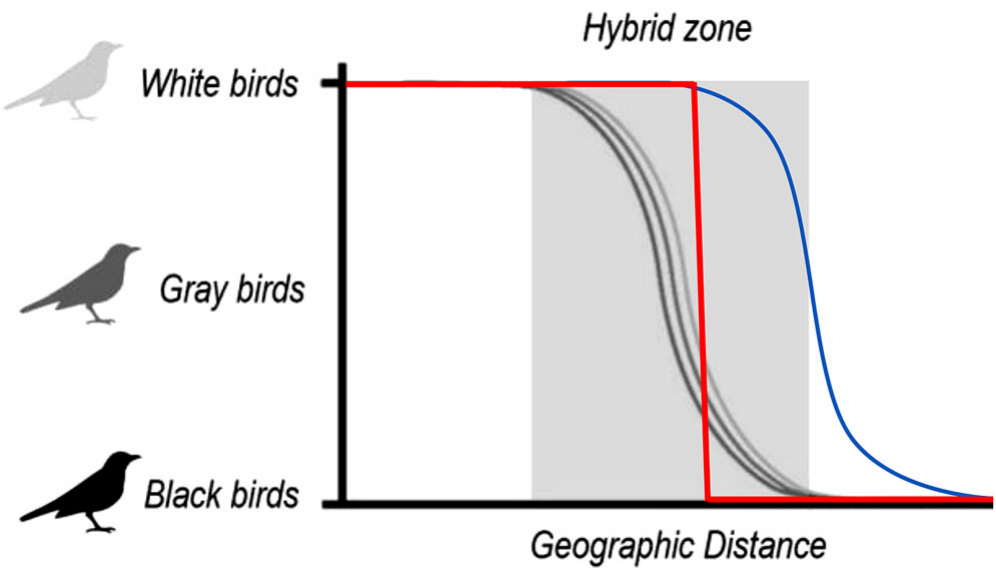
The shape of different clines within a hypothetical hybrid zone between a black and a white bird species. Most clines show a gradual transition (gray lines), suggesting neutral processes acting on the underlying genetic markers. The steep red cline points to strong selection against hybrids, whereas the blue cline indicates introgression from one species into the other.

The shape of the cline provides information on the strength of selection against hybrids. If the gray hybrids interbreed with each other and the parental species, there will be a variety of differently colored backcrosses. This will result in a smooth transition from white through different shades of gray to black: a wide cline. However, if the hybrids do not reproduce, there will be similarly colored gray birds in the narrow contact zone, resulting in a rapid transition from white to black plumage: a steep cline. Moreover, the position of the cline can provide insights into introgression dynamics. With no hybridization outside the hybrid zone, all clines should be centered on the midpoint of the hybrid zone. But if there is introgression from one species into the other, the cline center can be displaced into the distribution of one species. The displaced cline might represent a certain phenotype or genetic variant that confers an adaptive advantage.

In this issue, Del‐Rio et al. (2021) applied cline theory to a hybrid zone between the white‐breasted antbird (*Rhegmatorhina hoffmannsi*) and the harlequin antbird (*R. berlepschi*) in Amazonia. Their analyses revealed a narrow cline for the mitochondrial marker ND2, which was also displaced with respect to the clines for other traits and nuclear markers. The narrow mitochondrial cline suggests strong selection against hybrids. Given that mtDNA is inherited through the maternal line, it seems plausible that female hybrids suffer from decreased fertility or viability: a pattern in line with Haldane's Rule (Delph and Demuth 2016). The displaced position of the mitochondrial cline can be explained in several ways. It might be the outcome of one haplotype reaching fixation due to genetic drift or selection. Alternatively, the mtDNA might have failed to track the northward movement of the hybrid zone that is reflected in the other clines.

The hypothesis of hybrid zone movement is supported by a prediction that follows from a biological invasion scenario (Currat et al. 2008). Initially, the invading species (assumed to be *R. hoffmannsi*) will be outnumbered by the local species (*R. berlepschi*), resulting in hybridization. As the invasion proceeds, the invading species and previously produced hybrids will be part of the local population, thereby overturning the numerical imbalance. Consequently, hybrids will have a higher chance of backcrossing with the invading species, resulting in asymmetric introgression from local (*R. berlepschi*) into the invading species (*R. hoffmannsi*). And indeed, the researchers detected introgression in this direction. Why the mtDNA did not track the moving hybrid zone remains to be investigated, but might be related to the fitness reduction of female hybrids described above.

Associate Editor: K. Moore

Handling Editor: T. Chapman

## SUBMIT A DIGEST

Digests are short (?500 word), news articles about selected original research included in the journal, written by students or postdocs. These digests are published online and linked to their corresponding original research articles. For instructions on Digests preparation and submission,please visit the following link: https://sites.duke.edu/evodigests/
